# *Glycyrrhiza glabra* L. Extracts and Other Therapeutics against SARS-CoV-2 in Central Eurasia: Available but Overlooked

**DOI:** 10.3390/molecules28166142

**Published:** 2023-08-19

**Authors:** Murat Zh. Zhurinov, Alfira F. Miftakhova, Viktoriya Keyer, Zarina T. Shulgau, Elena V. Solodova, Maxat K. Kalykberdiyev, Arlan Z. Abilmagzhanov, Eldar T. Talgatov, Sauyk Ait, Alexandr V. Shustov

**Affiliations:** 1 “D.V. Sokolskiy Institute of Fuel, Catalysis and Electrochemistry” JSC, Almaty 050010, Kazakhstan; 2 Faculty of Chemistry and Chemical Technology, Al-Farabi Kazakh National University, Almaty 050040, Kazakhstan; 3 Laboratory for Genetic Engineering, RSE “National Center for Biotechnology”, Astana 010000, Kazakhstan; 4 Department of Biochemical Engineering, International Engineering Technological University, Almaty 050040, Kazakhstan

**Keywords:** antivirals, COVID-19, Cridanimod, Favipiravir, *Glycyrrhiza glabra*, Glycyrrhizin, influenza virus, licorice, phytochemicals, SARS-CoV-2 virus, Tilorone

## Abstract

In Central Eurasia, the availability of drugs that are inhibitors of the SARS-CoV-2 virus and have proven clinical efficacy is still limited. The aim of this study was to evaluate the activity of drugs that were available in Kazakhstan during the acute phase of the epidemic against SARS-CoV-2. Antiviral activity is reported for Favipiravir, Tilorone, and Cridanimod, which are registered drugs used for the treatment of respiratory viral infections in Kazakhstan. A licorice (*Glycyrrhiza glabra*) extract was also incorporated into this study because it offered an opportunity to develop plant-derived antivirals. The Favipiravir drug, which had been advertised in local markets as an anti-COVID cure, showed no activity against SARS-CoV-2 in cell cultures. On the contrary, Cridanimod showed impressive high activity (median inhibitory concentration 66 μg/mL) against SARS-CoV-2, justifying further studies of Cridanimod in clinical trials. Tilorone, despite being in the same pharmacological group as Cridanimod, stimulated SARS-CoV-2 replication in cultures. The licorice extract inhibited SARS-CoV-2 replication in cultures, with a high median effective concentration of 16.86 mg/mL. Conclusions: The synthetic, low-molecular-weight compound Cridanimod suppresses SARS-CoV-2 replication at notably low concentrations, and this drug is not toxic to cells at therapeutic concentrations. In contrast to its role as an inducer of interferons, Cridanimod is active in cells that have a genetic defect in interferon production, suggesting a different mechanism of action. Cridanimod is an attractive drug for inclusion in clinical trials against SARS-CoV-2 and, presumably, other coronaviruses. The extract from licorice shows low activity against SARS-CoV-2. At the same time, high doses of 2 g/kg of this plant extract show little or no acute toxicity in animal studies; for this reason, licorice products can still be considered for further development as a safe, orally administered adjunctive therapy.

## 1. Introduction

People in Kazakhstan, as in other countries, have experienced great moral and economic losses due to the recent (and still ongoing) pandemic of the SARS-CoV-2 coronavirus infection [1,2]. Ultimately, the incidence of clinically registered cases of SARS-CoV-2 in Kazakhstan has diminished because of vaccination and the development of population immunity upon infection [3]. However, at the beginning of the epidemic, when perspectives on vaccination were unclear, the control strategy employed mainly focused on enforcing quarantine and social distancing [4]. For those who contracted the disease, all available means of treatment were considered by medical professionals, with an emphasis on using substances that, at least potentially, could inhibit SARS-CoV-2 or boost immunity.

At present, despite the easing of this clinical burden, the SARS-CoV-2 virus continues to spread with new cases. It is expected that this virus will never disappear from the epidemiological circulation and will behave similarly to human seasonal coronaviruses. Thus, antivirals that are active against SARS-CoV-2 will be in demand, as hospitalized patients with aggravated disease will require pharmacological control for SARS-CoV-2 infection.

Potent SARS-CoV-2 inhibitors are present in the form of therapeutic virus-neutralizing monoclonal antibodies in the markets of developed countries. However, these drugs are expensive and not readily available in developing countries [5]. Among the small-molecule antivirals for the coronavirus, only a few have proved their clinical efficacy in registered clinical trials [6,7].

Apart from biopharmaceuticals, in the USA, only the drug Remdesivir has received the FDA’s unlimited approval as an etiotropic therapy [8,9]. Another drug, Paxlovid, received Emergency Use Authorization (EUA), but this EUA was withdrawn due to the drug’s inefficiency against newer viral strains. Many countries in the Old World have approved the anti-influenza drug Favipiravir for the treatment of SARS-CoV-2 infection [10,11,12]. In most of Eurasia, at the height of the epidemic, Favipiravir was employed as the standard-of-care drug for seriously ill patients in hospital settings.

The aim of this study was to evaluate the activity of drugs that were available in Kazakhstan during the acute phase of the epidemic against SARS-CoV-2. Here, antiviral activity is reported for three registered drugs: Favipiravir, Tilorone, and Cridanimod (CMA) (which are registered in the national pharmacopeia and recommended for the treatment of viral infections). An extract derived from plant licorice (*Glycyrrhiza glabra*) was also incorporated into this study because of the numerous publications on the antiviral activity of licorice phytochemicals, as well as the abundance of this plant in Kazakhstan and its potential for the rapid production of licorice-derived cures [13,14]. From the results of this study, it appeared that that potential therapeutics against SARS-CoV-2 were available to the population and physicians even during the initial phase of the epidemic, among which Cridanimod has attracted special attention for its high activity in inhibiting SARS-CoV-2 replication and its low toxicity.

## 2. Results

### 2.1. Producing and Testing Licorice Extract

Table 1 presents the contents of the extractives when using ethyl alcohol as a solvent in the process of extraction of bioactive compounds from licorice roots.

Water is an efficient solvent used to extract phytochemicals from *G. glabra* roots. However, using pure water also results in the extraction of admixtures, such as polysaccharides and proteins. The extracts obtained with 90% ethanol contained water-insoluble ballast substances such as gums or lipids. Considering a balance between quantity and purity, we elected to use a 1:1 ethanol/water mixture as the extraction solvent.

**Figure 1:**
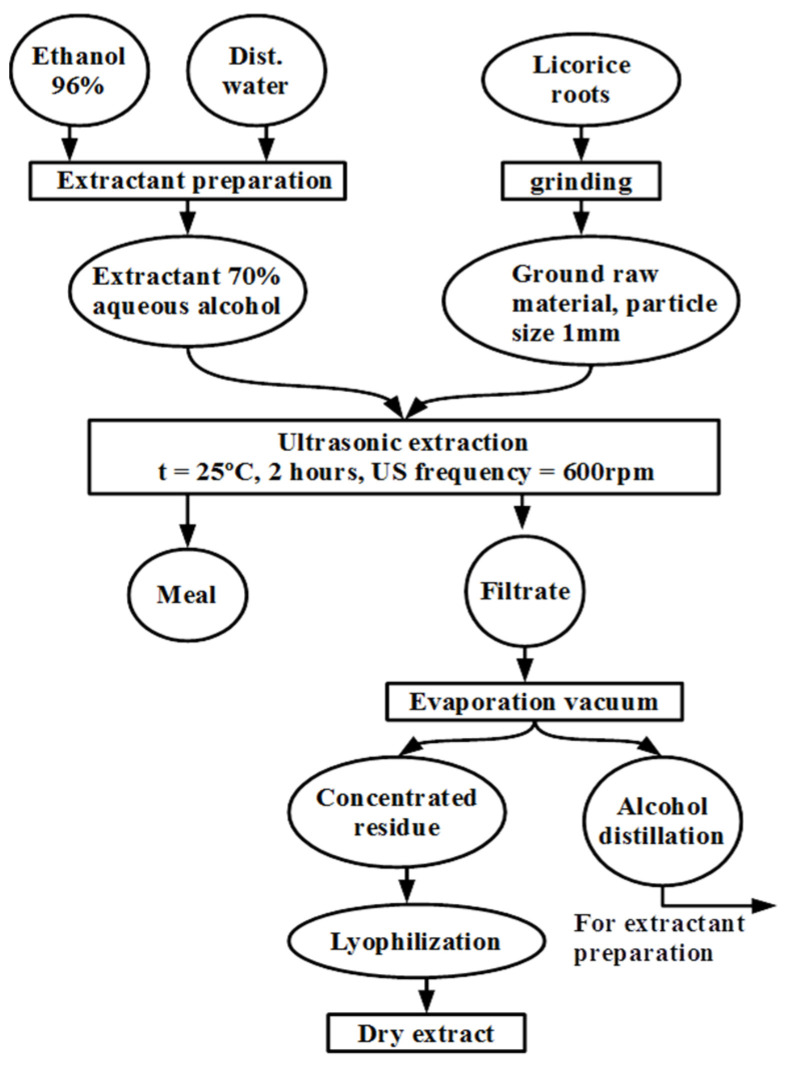
Diagram of the process of obtaining a dry extract from licorice roots.

The authors developed a method for producing extracts from *G. glabra* roots, which is depicted in Figure 1.

**Figure 2:**
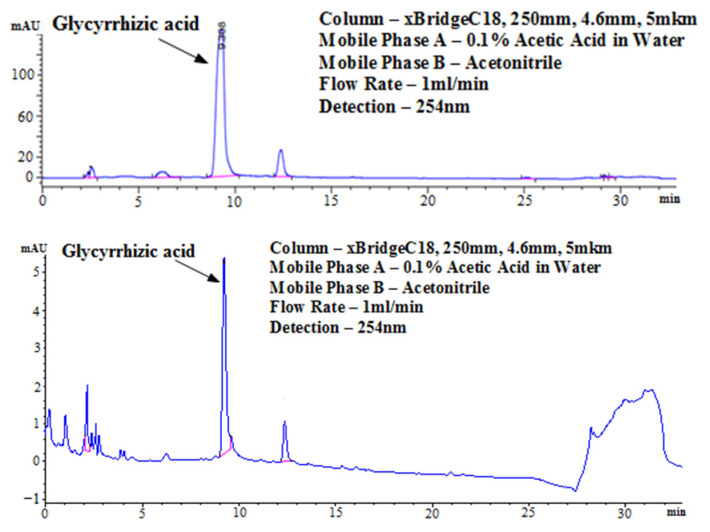
HPLC chromatograms of the Glycyrrhizin standard (**upper panel**) and obtained licorice extract (**lower panel**).

The purity of the obtained extract was analyzed with HPLC using pure Glycyrrhizin as a purity standard (Figure 2). Glycyrrhizin provides a distinctive chromatogram during HPLC, which is characterized by a major peak at 9.2–9.4 min and a shoulder peak at 12.3 min. Comparing the obtained extract with the standard reveals the presence of only 18 small peaks in addition to the peaks characteristic of the main component. This licorice extract was used in our experiments to measure inhibitory activities against SARS-CoV-2 and influenza viruses.

### 2.2. Antiviral Activity against SARS-CoV-2: CMA Shows the Highest Activity

**Figure 3:**
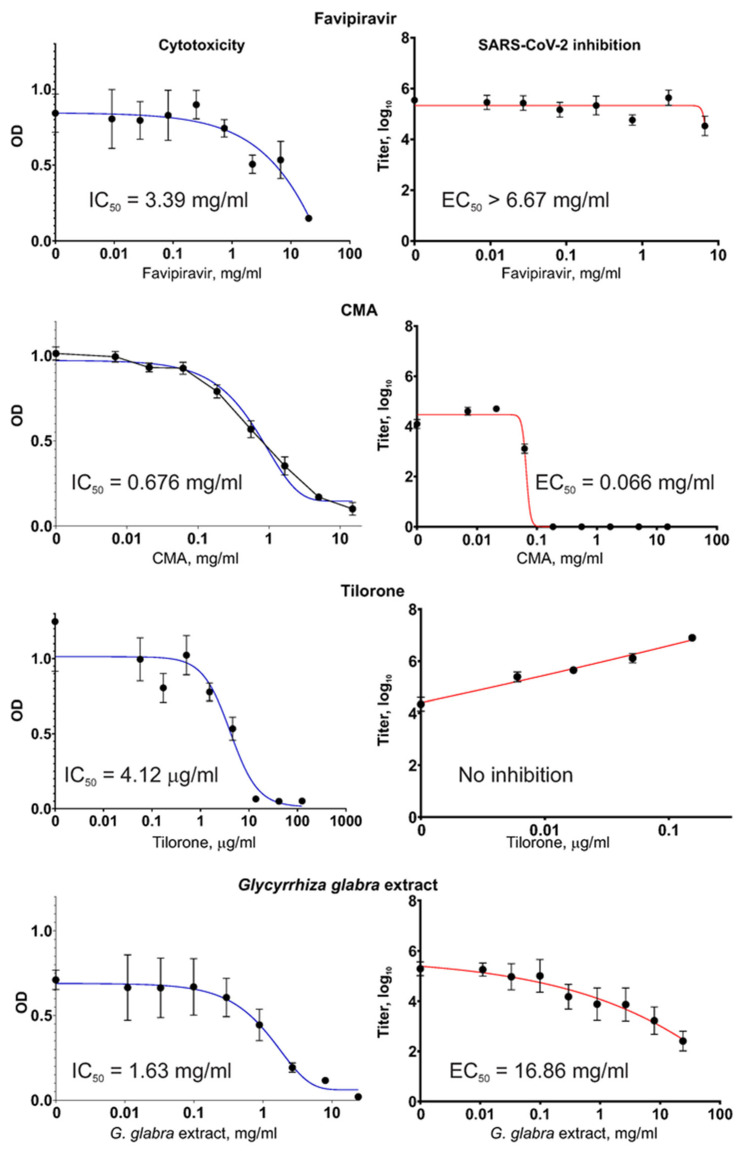
Four substances, Favipiravir, CMA (Cridanimod), Tilorone, and a *G. glabra* extract, were used to measure cytotoxicity in Vero E6 cells and their potential to inhibit SARS-CoV-2 replication in cell cultures. (**Left panels**): As described in the Materials and Methods, 50% inhibitory concentrations (IC_50_) were measured in 96-well-plate cultures. Tilorone is distinguished from other substances by its much higher cytotoxicity. (**Right panels**): As described in the Materials and Methods, 50% effective concentrations (EC_50_) were measured using the virus yield reduction assay with cell cultures grown in P100 dishes. No virus inhibition was found for Tilorone at non-cytotoxic concentrations. On the contrary, for the distantly related compound CMA, EC_50_ = 66 μg/mL, which is the lowest value, indicating that it has the highest activity among the tested substances.

Different substances, including the *G. glabra* extract and registered drugs were studied for their inhibitory activity against the SARS-CoV-2 coronavirus and their own cytotoxicity. The results of these measurements are shown in Figure 3.

Of the four tested substances, only Tilorone showed notably high cytotoxicity, with an IC_50_ close to the range of therapeutically achievable concentrations. The other substances, including the *G. glabra* extract, were non-cytotoxic within a range of therapeutically meaningful concentrations.

In our experiments, the Favipiravir compound did not diminish the SARS-CoV-2 virus titers when the virus was grown in Vero E6 cells, even when Favipiravir was present at very high concentrations of >6 mg/mL. This observation was not the result of a mistake during the preparation of the Favipiravir solutions or other possible sources of error (e.g., when handling the fake drug tablets) because the same Favipiravir solutions showed a high capacity to suppress the influenza virus (described in Section 2.3. Antiviral Activity against Influenza Virus).

The Tilorone drug showed no reduction in the level of SARS-CoV-2 titers in the Vero E6 cell cultures when used within the non-cytotoxic range of concentrations; rather, the titers increased proportionally to the Tilorone concentration, indicating a stimulating effect. At the highest non-cytotoxic concentration of 154 μg/mL, the obtained SARS-CoV-2 titer was 161× the value of the titer from a control culture with no addition of Tilorone (*p* < 0.001). Microscopic examination of the virus-producing cultures during the VYRA test revealed that at this concentration (slightly below the cytotoxic concentration), Tilorone actually protects cells from death resulting from the infection-induced cytopathic effect (CPE).

In contrast, CMA, which is a distinct compound but generally included in the same pharmacological group as Tilorone, efficiently inhibited SARS-CoV-2 replication. For CMA, the EC_50_ = 66 μg/mL (i.e., 260 μM), which indicates that it possesses the highest antiviral activity among the tested substances.

The obtained *G. glabra* extract showed virus inhibition, which was also concentration-dependent. However, the EC_50_ was high. It is worth mentioning that IC_50_ and EC_50_ were measured in tests with different set-ups for the infected cultures. In the VYRA test, some cells survived and produced the virus even when the drug’s concentration was above IC_50_. This explains why the values of IC_50_ and EC_50_, which are medians computed from regression curves, may show a situation where EC_50_ > IC_50_.

### 2.3. Antiviral Activity against Influenza Virus

**Figure 4:**
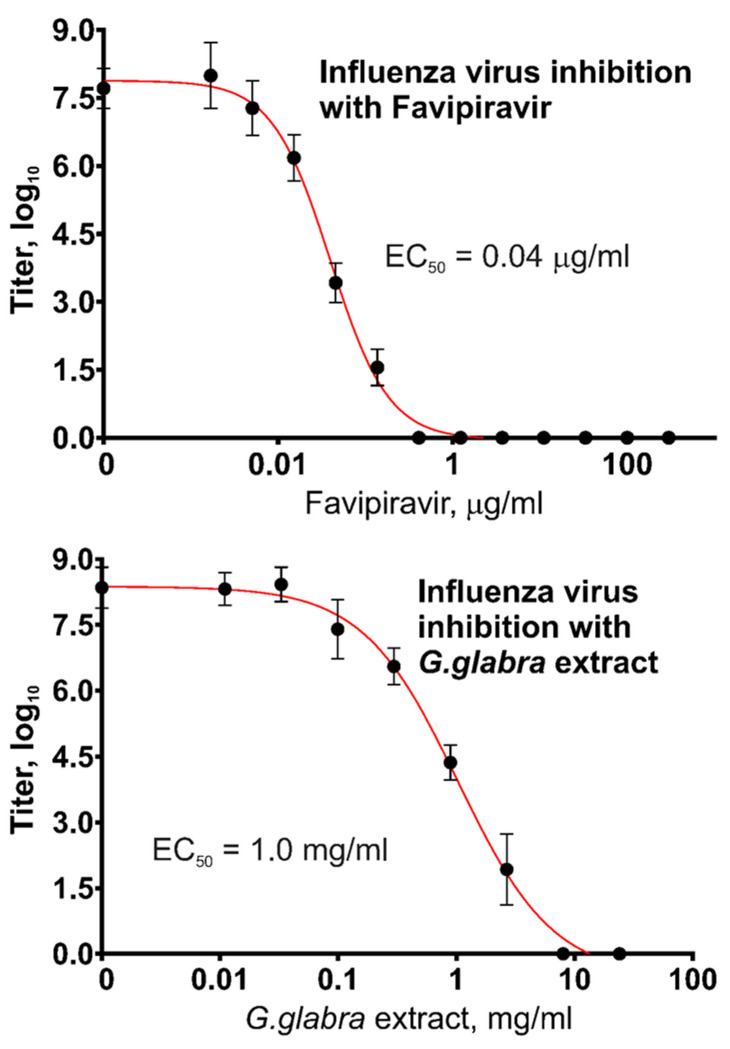
Antiviral activities of Favipiravir and *G. glabra* extract against type A influenza virus in the VYRA test.

The results obtained in this work on the use of Favipiravir against SARS-CoV-2 showed no activity but confirmed that the initial source of this chemical (drug tablets) contains the active substance, Favipiravir, in the stated amounts. For confirmation, we produced a similar VYRA test with a different virus, namely, influenza type A/H1N1 virus, because the published literature describes the high sensitivity of the influenza virus to Favipiravir. Figure 4 presents the measurement results regarding the antiviral activity of two substances, Favipiravir and *G. glabra* extract, against type A influenza virus. The same Favipiravir solution and the same plant extract were used, and the results regarding their effects against SARS-CoV-2 are shown in Figure 3.

Favipiravir demonstrated a remarkable ability to inhibit influenza virus replication, with EC_50_ = 40 ng/mL. The inhibition of influenza virus by the *G. glabra* extract was also observed, though at higher concentrations.

### 2.4. Acute Toxicity Testing

The plant extract was dried, and the dry material was resuspended in a minimal volume of drinking water and administered intragastrically to mice using doses of 2 g (dry extract) per 1 kg (mouse weight). This was the maximum technically achievable dose for intragastric administration, given the limited solubility of the extract. The mice in the experimental groups did not show changes in body weight during the two weeks of observation. No changes were observed in the behavior, bodily appearance, or motion activity of the animals. No deaths were registered in either group. A biochemical study of the animals’ urine, carried out 1 day after administering the extract, revealed an increase in the number of erythrocytes; however, at the next testing step after 1 week, the erythrocytes in the urine had returned to the same numbers as the control. Moreover, 1 day after administering the extract, glucose appeared in the urine, but after 1 week, glucose was normal. No other deviations from the norm were evident in the urine. The appearance and weights of the internal organs in the experimental groups were normal and not statistically different from those in the control mice. These results indicate a low or absence of acute toxicity of the obtained *G. glabra* extract.

## 3. Discussion

Respiratory-transmitted viruses can cause pandemics and inflict significant damage, a truth that all of humanity has recently witnessed. At the beginning of the SARS-CoV-2 epidemic in Central Eurasia, local healthcare systems appeared almost defenseless, specifically in rescuing critically ill patients. Those individuals were treated with antivirals available at hand, even though, in some cases, their specific activity against SARS-CoV-2 had not been confirmed or there was a lack of evidence of their clinical efficacy.

Presently, therapeutic monoclonal antibodies neutralizing the SARS-CoV-2 virus are available and efficient; four brand names have EUA in the USA [15]. However, these biopharmaceuticals are practically nonexistent in developing countries.

At the beginning of the pandemic, there were no clinical drugs developed specifically as inhibitors of coronaviruses. Analyzing the response during the acute period of the epidemic and evaluating the effectiveness of early pharmacotherapy for the SARS-CoV-2 infections are important preparation steps for future epidemics, especially considering that these events may repeat themselves because the biological nature of an epidemic virus is relatively unpredictable, and there will probably be no specific drugs to combat the virus in question.

This paper describes the testing of three registered antivirals used in Kazakhstan during the onset of the epidemic and compares them to an antiviral that has been considered for commercial production and is a plant extract.

The three pharmacopoeial drugs discussed in this work belong to different classes. One was used in the tests because, based on its chemical nature, it could have a direct inhibitory effect on SARS-CoV-2. The other two drugs were originally approved as inductors of endogenous interferon (IFN). However, later, as shown by other authors and in this work, the antiviral effect of these two drugs is not necessarily mediated by the induced IFN. One prospective plant extract was tested in the described experiments because its associated technology has shown potential for the domestic production of antiviral pharmaceuticals in Kazakhstan for the first time. The need to reduce our dependence on imported pharmaceuticals has been acknowledged in this country for a long time, and therefore, phytochemicals are an important topic for consideration in industry.

Favipiravir is a nucleoside analog introduced into the market by Fujifilm Toyama Chemical (Japan) as an anti-influenza drug. Favipiravir is highly active against influenza virus types A, B, and C (EC_50_ values 0.014–0.55 μg/mL) [16]. Importantly, Favipiravir efficiently suppresses the replication of mutant influenza viruses, which are resistant to more widely used neuraminidase inhibitors. However, the efficacy of Favipiravir against SARS-CoV-2 is controversial. One study found that for Favipiravir, EC_50_ = 61.88 μM against SARS-CoV-2 [17], while other papers reported higher EC_50_ values, e.g., 207.1 μM [18] or >500 μM (i.e., >78 μg/mL) [19]. Registered clinical trials showed marginal benefits of Favipiravir during the treatment of SARS-CoV-2 patients [20]. Favipiravir was once included in standard-of-care protocols for the treatment of severe SARS-CoV-2 infections in Kazakhstan, but its low clinical efficacy resulted in its exclusion from the recommended therapeutic schemes. The presented results confirm that Favipiravir is an inefficient inhibitor of SARS-CoV-2. However, we also confirmed that Favipiravir is a highly efficient inhibitor of the influenza virus.

Tilorone and Cridanimod (CMA) are synthetic low-molecular-weight (Low-Mw) molecules, being tricyclic aromatic molecules in the classes of fluorenones and acridones, respectively. These compounds were noted for their ability to induce IFN in mice [21]. For this reason, in countries in which they are registered, Tilorone and CMA are still placed in the pharmacological group of “immunomodulators; IFN inducers; antiviral agents”. They are predominantly used as tablets for intragastric administration. At present, Low-Mw IFN-inducers are not in clinical use as antivirals in modern Western medicine, but Tilorone had FDA approval in the mid-1970s. In fact, convincing evidence has been published showing that Tilorone and CMA, at therapeutic dosages, do not induce a significant rise in the level of circulating IFN in humans or animal species distant from murides [22,23,24,25]. Thus, the mechanisms of these drugs’ therapeutic action in humans have not yet been deciphered. However, ample literature has been published proving that Tilorone and CMA have antiviral activity in vitro. More importantly, both drugs have proven clinical efficiency in the treatment and prophylaxis of diverse viral infections in humans. The scientific literature on the clinical efficiency of both Tilorone and CMA as antivirals includes numerous Russian and Chinese sources, possibly because these drugs have been registered and in use for a long time in a large part of Eurasia [26]. Tilorone and CMA exert antiviral activity in experimental conditions where there is no IFN induction, e.g., in Vero cells. It has been suggested that Tilorone and CMA can induce an IFN-independent antiviral state [26]. Yet, the intracellular molecular targets with which these drugs interact have not been identified.

In this work, we investigated the ability of Tilorone and CMA to inhibit SARS-CoV-2 replication in Vero E6 cells. The preliminary assumption was that Tilorone and CMA, as representatives of the same pharmacological group, would exhibit similar effects on viral replication. It turned out that these two drugs differ in their effects. Tilorone is highly cytotoxic, while CMA is not toxic to cell cultures at pharmacologically relevant concentrations.

Two effects were observed for Tilorone: firstly, this drug has a pronounced concentration-dependent (up to the concentration of its own cytotoxicity) stimulating effect on SARS-CoV-2 replication in Vero E6 cells; secondly, in a certain concentration range, Tilorone protects virus-infected cells from death resulting from the virus-induced cytopathic effect. Similar observations were made by the Ekins group [27], who also worked on Vero E6 cells and found no inhibition of SARS-CoV-2 by Tiloron (Figure 2 in ref. [27]). In fact, the authors of this work found a use for this effect in increasing SARS-CoV-2 titers in the presence of sub-cytotoxic concentrations of Tilorone. The authors intentionally added Tilorone (154 μg/mL) to SARS-CoV-2-producing Vero E6 cultures to obtain >100-fold larger amounts of the antigen (compared to the control cultures without the drug) during the preparation of the viral antigen for immunological experiments.

Among our main findings is that CMA is active against SARS-CoV-2, with the therapeutically relevant EC_50_ = 66 μg/mL. It is interesting to speculate on the possible mechanism of CMA’s antiviral action. The published studies demonstrated that CMA quickly penetrates through the cell membrane and binds to unidentified cellular components in proximity to the nuclear envelope, and it shows high intracellular tropism [28]. This complicates the collation of the pharmacokinetics with the observed efficiency because a large fraction of the drug, when bound intracellularly, makes irrelevant any comparison of the maximum serum concentration (C_max_) with EC_50_. It was postulated that CMA acts on mitochondria because CMA reduces mitochondrial membrane potential [28]. It has long been known that a fraction of cellular mitochondria are connected to the endoplasmic reticulum (ER) and located in the perinuclear space [29]. In this regard, novel, interesting, and relevant data were published, showing that SARS-CoV-2 replicative organelles (double-membrane vesicles, DMV) are actually produced from the membranous structures that connect the mitochondria with the ER [30]. A comparison of our findings and the published data allows us to propose a tentative hypothesis that CMA affects perinuclear mitochondria and somehow interferes with the generation of DMV from the ER–mitochondrial membranes.

The SARS-CoV-2 virus is not the only representative of the genus *coronavirus* of high importance because there are different species that are also important human or veterinary pathogens. In this regard, it would be interesting to test the antiviral action of CMA against different coronaviruses. Our study is one in a series that points to CMA’s role as an attractive drug for inclusion in clinical trials against infections caused by various coronaviruses.

The data presented here allow us to postulate that the mechanisms of action and intracellular molecular targets of Tilorone and CMA are different. Both drugs exert biological activities in Vero E6 cells, which do not produce type-I IFNs [31]. Thus, again, our observations are in favor of the notion that the antiviral action of Tilorone and CMA is different, not inducing IFNs or IFN-mediated signaling.

Medicinal plants are known to be rich sources of antiviral agents, among which some of the substances also have low toxicity, which is advantageous for their possible therapeutic usage. Licorice (*Glycyrrhiza glabra* L.) is well known for its antiviral activity against a variety of viruses [32,33]. Licorice is endemic in Kazakhstan and may be considered as a source plant for phytochemical production. The main antiviral components of licorice are triterpenoid saponins such as Glycyrrhizin (GN) and 18β-glycyrrhetinic acid (GA). In cell culture experiments, GN and GA showed antiviral activities against a variety of viruses: hepatitis A, B, and C; HIV; Coxsackie; influenza viruses (H1N1, H5N1); and SARS-CoV [34,35,36].

The mechanisms of the antiviral activities of triterpenoids remain understudied. It was reported that GN interferes with the early steps of the viral reproductive cycle, the binding of the virus to its receptor, and its internalization via endocytosis and uncoating in the cytoplasm. Two goals of our study were to develop a method for producing licorice extracts and study their activity against SARS-CoV-2 in cell cultures. The reported method allows one to obtain extracts, which, according to the analysis, contain GN of high purity.

For a given substance, its antiviral activity in tests conducted in vitro and its efficiency in the clinic may differ for obvious reasons. In this work, we identified one compound (CMA) that is non-toxic and shows high activity against SARS-CoV-2, with an EC_50_ close to the therapeutically achievable concentrations. CMA has been a registered drug in the authors’ country for a long time, and it is widely available in local pharmacies. It is possible that at the beginning of the SARS-CoV-2 epidemic in Central Eurasia, an efficient antiviral was at hand but remained overlooked.

## 4. Materials and Methods

### 4.1. Registered Antivirals

Favipiravir (6-fluoro-3-hydroxypyrazine-2-carboxamide), CMA (with the international nonproprietary name Cridanimod: 10-carboxymethyl-9-acridanone methylglucamine salt), and Tilorone (2,7-Bis[2-(diethylamino)ethoxy]-9-fluorenone dihydrochloride) are registered drugs in Kazakhstan. These drugs were purchased from a pharmacy as tablets containing the following amounts of active substances: 200 mg Favipiravir (Glenmark Pharmaceuticals Ltd., Mumbai, India), 150 mg CMA (LLC “Polisan NTFF”, St.-Peterburg, Russia), and 125 mg Tilorone (LLP “Viva Pharm”, Almaty, Kazakhstan). The tablets were crushed in a mortar, and the soluble compounds were dissolved in a growth medium (DMEM + 1% heat-inactivated FBS) to obtain stock concentrations of 10 mg/mL for Favipiravir and 20 mg/mL for other substances. The suspensions of crushed tablets were placed briefly in a water bath (50 °C) to ensure the dissolution of the active substances. These drugs are water-soluble at a neutral pH at the indicated concentrations. The solutions were clarified via centrifugation and sterilized via filtering.

### 4.2. Collection and Preparation of Plant Raw Materials

The collection and preparation of the plant materials of *Glycyrrhiza glabra* L. *roots* were performed in accordance with the Good Agricultural and Collection Practices For Medicinal Plants (GACP). The raw materials of the *Glycyrrhiza glabra* L. *roots* were collected in late autumn during the middle of November 2021 in the Almaty region (43°15 N, 76°57 E), specifically during the period of the maximum accumulation of biologically active compounds. The roots were dug up, but at least one-quarter of the rhizomes were left in the soil. After that, the reaming earth was cleaned from the original licorice roots, which were then washed under cold water. Then, they were cut into smaller pieces with special secateurs and dried at the “D.V. Sokolsky Institute of Fuel, Catalysis and Electrochemistry”, JSC, in the shade on special frames at an ambient temperature of 25 ± 5 °C for a week. The *Glycyrrhiza glabra* L. *roots* were subjected to additional grinding in a mill until a size of no more than 3–5 mm was obtained. The particle size was controlled via sieving.

### 4.3. Producing Extracts from Glycyrrhiza glabra L. roots

Bioactive compounds with potential antiviral activity were extracted from the plant material (*G. glabra*) using ultrasound-assisted extraction with a PS-06A ultrasonic bath. The dried roots were ground into ~1 mm pieces. The weighted portion (100 g) was dispersed in a water–ethanol mixture (500 mL of 50% ethanol in water). The flask was placed in the ultrasonic bath and treated with 40 kHz for 1 h at 30 °C. The extraction cycle was 30 min of ultrasound action and 30 min of mixing on a rotary mixer, and this cycle was repeated twice. The extract was filtered and concentrated using a rotary evaporator, a Stuart RE400 (Cole-Parmer Ltd., Saint Neots, UK) set at 65 °C until the volume was reduced to 1/3 of the original volume.

### 4.4. High-Performance Liquid Chromatography

An Agilent 1100 liquid chromatography system equipped with an xBridge C18 column (250 mm, 4.6 mm, 5 µm) and UV detector set to three wavelengths (210, 254, and 360 nm) was used to analyze the contents in the plant extracts. The extracts were dried and weighed. Solutions with a concentration of 100 mg/L were prepared in a mixture of acetonitrile–water (1:1) and injected (10 µL or 100 µL) using an autosampler. Mobile phase A was acetic acid 0.1% in water. Mobile phase B was pure acetonitrile. The other separation conditions were a flow rate of 1 mL/min and a temperature of 30 °C.

### 4.5. Cell Culture and Virus Strain

Vero E6 (ATCC CRL-1586) and MDCK (ATCC CCL-34) cells were obtained from the National Center for Biotechnology collection (Nur-Sultan, Kazakhstan). The cell lines were grown in DMEM with high glucose (Lonza BE12-604 F/U1) supplemented with 10% FBS (Gibco Cat# 16000-044), 2 mM L-glutamine, 1% MEM vitamin solution (ThermoScientific (Waltham, MA, USA) Cat# 11120052), 1% non-essential amino acids (ThermoScientific Cat# 11140050), penicillin (100 U/mL) and streptomycin (100 µg/mL).

The used coronavirus strain was obtained via isolation from a clinical sample and named hCoV-19/Kazakhstan/20679/2020. This virus’ genome was sequenced and deposited in the GISAID database (https://www.gisaid.org/, accessed on 17 July 2023) with the number EPI_ISL_454501 [37]. This coronavirus strain belongs to the B1 phylogenetic line, the isolates of which were frequent in the first half of 2020. The A/Puerto Rico/8/34(H1N1) influenza virus strain is maintained in the NCB’s collection.

### 4.6. Virus Stocks

Vero E6 cells for the culturing of SARS-CoV-2 or MDCK cells for the influenza virus were seeded in P100 dishes at 2 × 10^6^ cells per dish. At 90% confluence, the media were changed to a reduced-serum medium (containing 1% heat-inactivated FBS). The cultures were infected using a multiplicity of infection (MOI) of 0.01. Incubation proceeded for 72 h, after which the virus-containing media were collected. The media were clarified via centrifugation, aliquoted, and stored at −80 °C [38].

### 4.7. Virus Titering

A method based on end-point dilutions of samples (Reed–Muench) was used [39]. Permissive cells were seeded in 96-well plates (37,500 cells per well). Serial dilutions were obtained using DMEM + 2% heat-inactivated FBS as a diluent. Eight tenfold dilutions were prepared (1:10 to 1:10^8^). The dilutions were distributed in the plates’ long rows. On each plate, one vertical row, 12, was filled with the medium without the virus and served as an uninfected control. The plates were incubated for 3–4 days until the virus-induced cytopathic effect (CPE) became visible. Wells with the CPE were counted per row and used to compute the titers using the Reed–Muench scheme [40].

For some measurements during the experiments on the plant extracts, the titers were measured using a plaque assay.

### 4.8. Cytotoxicity Test

The cytotoxicity of tested substances was measured by determining the half-maximal inhibitory concentration, IC_50_, i.e., the concentration at which the tested compound reduces the number of live cells in the culture by 50% [41].

Vero E6 or MDCK cells were seeded in 96-well plates at 20,000 cells per well. The plates were incubated overnight. The next day, the media were changed to a fresh medium (DMEM + 1% FBS, 100 µL per well). The medium was removed from the wells of row H (wells 1–10). Wells 1–10 of row H were filled with 150 µL aliquots of the drugs (at their stock concentrations or different defined concentrations) in DMEM + 1% FBS. Aliquots of 50 µL were picked from the wells of row H and transferred to row G with mixing. The transfer was continued until rows A–H (vertical rows 1–10) were filled with the drug-containing medium. The between-row dilution factor was 1/3. In each plate, vertical rows 11 and 12 were not supplemented with drugs so as to be used as native controls.

The plates were incubated for 3 days in a CO_2_ incubator. Microscopic observation of the cultures was used to control for the cytopathic effect (CPE). At the end of the experiment, the remaining live cells were stained with either neutral red or nitroblue tetrazolium (MTT). Then, 100 µL aliquots of fresh media containing 0.011% neutral red (Sigma Cat. N4638) were added to all the wells. After 2 h of incubation, the media were completely removed, and the wells were gently rinsed with PBS and dried. Acetic acid (1% solution in water) was added to wells to allow the dye to re-dissolve. The optical absorbance was measured using a plate reader at 540 nm. Alternatively, 20 μL aliquots of MTT (3 mg/mL, Sigma Cat. M2128) solution in a medium base was added into the wells. The plate was incubated for 3 h. The medium was removed, taking care not to dislodge the stained cells. Then, 100 μL of DMSO with 1% acetic acid was added to the wells to dissolve the formazan. The optical density was determined at 595 nm. The experiments were performed in triplicates.

### 4.9. Antiviral Activity

Antiviral activity was measured as the 50% efficient concentration (EC_50_), which is a concentration that inhibits half the virus yield produced after the infection of a culture with a small infectious dose. The virus yield reduction assay (VYRA) is a two-step procedure: in the first step, the virus is grown in cultures in the presence of different concentrations of a tested substance; in the second step, the titers of viral progeny are determined using the titration procedure described above. Here, permissive cells were seeded in P100 dishes at 10^6^ cells per dish. Nine dishes were seeded in this way for each tested substance. Once the majority of the cells had been attached, the growth medium was changed to DMEM + 1% FBS (10 mL per dish). One dish was left without the addition of the tested substance. To the other eight dishes, the substance was added to produce a range of concentrations with a step dilution factor of 1/3. In the majority of the experiments, the range of working concentrations was from 125 μg/mL to 0.057 μg/mL. Immediately upon the addition of the substance to the cultures, an infectious inoculum containing SARS-CoV-2 or the influenza virus was added to the cultures. In all the experiments, the multiplicity of infection (MOI) was 0.01. The cultures were incubated for 3 days, after which the media were collected, and the aliquots were taken and stored at −80 °C. The VYRA experiments were performed in three biological replicates for each substance. The viral titers were determined and used to plot titer vs. substance concentration plots.

### 4.10. Data Processing

Graphs for the cytotoxicity and antiviral activity tests were produced in GraphPad Prism (GraphPad Inc., San Diego, CA, USA). Four-parameter non-linear regression was used to compute the half-maximal effective concentration EC_50_ and inhibitory concentration IC_50_.

### 4.11. Animal Studies for Acute Toxicity

Outbred CD-1 mice were bred in the NCB’s animal house. Mice of both sexes weighing 26–32 g were used for this work. The tested substances were administered via the intragastric route. The preliminary studies showed low or absent acute toxicity; hence, for the final experiments, the highest technically possible dose of 2 g (substance) per 1 kg (body weight) was used. The dried extracts were suspended in sterile drinking water, and the resulting solution was administered *ex tempore*. The experimental and control groups each included 12 mice (6 females and 6 males). Upon administering the substance or placebo, the mice were monitored for changes in body weight, overall appearance and behaviors, motion and feeding activity, the presence and nature of convulsions, the ability to react to stimuli, the frequency and depth of respiratory movements, and the state of the mucosa and skin. One day and one week after administering doses, the mice’s urine biochemistry was studied using an automatic analyzer, Uripolian-5A (Biosensor AN, Chernogolovka, Russia). At the end of the experiment, the mice were euthanized via CO2 asphyxia, and the internal organs were extracted and weighed. Comparison of blood tests and organ weights in experimental and control animals are given in Supplementary Materials Tables S1–S4.

## 5. Conclusions

Pharmaceutical inhibitors with proven activity against the SARS-CoV-2 virus are lacking in Central Eurasia. In this report, we outline the results of tests of three registered drugs that were used to treat patients during the epidemic for their SARS-CoV-2 virus inhibitory activity. Favipiravir showed little activity. On the contrary, CMA (Cridanimod) showed a notably low EC_50_ against SARS-CoV-2, justifying further testing in clinical trials against various infections caused by coronaviruses. The extract from licorice had low activity against SARS-CoV-2. However, it showed little or no acute toxicity in animal studies. For this reason, licorice extract has the potential for the development of orally administered adjunctive therapies.

## Figures and Tables

**Table 1:** Yield of substances from *Glycyrrhiza glabra* L. when using various extraction solvents.

Solvent	The Content of Extractives, %
90% ethyl alcohol	43.0
70% ethyl alcohol	38.0
50% ethyl alcohol	45.0
water	46.0

Note—Ultrasound-assisted extraction, raw material/extraction solvent ratio 1:9.

## Data Availability

The data that support the findings of this study are available from the corresponding author upon reasonable request.

## Supplementary Materials

The following supporting information can be downloaded at: https://www.mdpi.com/article/10.3390/molecules28166142/s1, Table S1: Effect of GGE on mouse body weight with single intragastric administration at a dose of 2 g/kg of mouse body weight; Table S2: GGE for the urine biochemical values one day after the administration of GGE with single intragastric administration at a dose of 2 g/kg of mouse body weight; Table S3: Effect of GGE on the urine biochemical values one week after the administration of GGE with single intragastric administration at a dose of 2 g/kg of mouse body weight; Table S4: Effect of GGE on the weight of internal organs of mice with single intragastric administration at a dose of 2 g/kg of mouse body weight.
