# Supplementary material for: Glycyrrhiza glabra L. Extracts and Other Therapeutics against SARS-CoV-2 in Central Eurasia: Available but Overlooked
Source: Molecules. 2023 Aug 19;28(16):6142. doi: 10.3390/molecules28166142 (PMC10458004; doi:10.3390/molecules28166142)
Supplement: Supplementary file 1 [file molecules-28-06142-s001.zip › molecules-2500309-supplementary.pdf]

molecules

MDPI

Supplementary Materials

Table S1: Effect of GGE on mouse body weight with single intragastric administration at a dose of 2 g/kg of mouse body weight.

Study Group	Initial body weight of mice, g	Mice body weight after 1 week of administration of Dry Extract of Licorice Roots, g	Mice body weight after 2 weeks of administration of Dry Extract of Licorice Roots, g
1 group, Dry Extract of Licorice Roots, ♂, n=6	32.4±0.8<br>p=0.5541	33.9±1.7<br>p=0.3961	35.4±1.5<br>p=0.4287
2 group, control, ♂, n=6	31.8±0.8	32.0±1.3	33.7±1.5
3 group, Dry Extract of Licorice Roots, ♀, n=6	29.3±1.5<br>p=0.4511	31.9±1.2<br>p=0.5066	31.5±1.2<br>p=0.2331
4 group, control, ♀, n=6	30.8±1.2	31.9±1.3	33.5±1.0

Table S2: GGE for the urine biochemical values one day after the administration of GGE with single intragastric administration at a dose of 2 g/kg of mouse body weight;

Study Group	Test parameters				
	Red Blood Cells, Units/μL	Ketones, mmol/L	Protein, g/l	Glucose, mmol/L	pH, units
1 group, GGE, ♂, n=6	3/6 – 25 Units/μL<br>2/6 – 50 Units/μL<br>1/6 – 250 Units/μL	6/6 – negative	6/6 – 0.1 g/l	3/6 – negative<br>1/6 – 2.8 mmol/L<br>2/6 – 14 mmol/L	6.0±0.0<br>(6/6–6.0)
2 group, control, ♂, n=6	6/6 – negative	6/6 – negative	4/6 – 0.1 g/l<br>2/6 – 0.3 g/l	6/6 – negative	6.0±0.0<br>(6/6–6.0)
3 group, GGE, ♀, n=6	3/6 – negative<br>2/6 – 10 Units/μL<br>1/6 – 25 Units/μL	6/6 – negative	4/6 – 0.1 g/l<br>2/6 – 0.3 g/l	3/6 – negative<br>2/6 – 2.8 mmol/L<br>1/6 – 5.6 mmol/L	6.0±0.0<br>(6/6–6.0)
4 group, control, ♀, n=6	6/6 – negative	6/6 – negative	4/6 – 0.1 g/l<br>2/6 – 0.3 g/l	6/6 – negative	6.0±0.0<br>(6/6–6.0)
Note: n – number of animals in a group					

Table S3: Effect of GGE on the urine biochemical values one week after the administration of GGE with single intragastric administration at a dose of 2 g/kg of mouse body weight.

Study Group	Test parameters				
	Red Blood Cells, Units/μL	Ketones, mmol/L	Protein, g/l	Glucose, mmol/L	pH, units
1 group, GGE, ♂, n=6	4/6 – 10 Units/μL<br>1/6 – 25 Units/μL<br>1/6 –50 Units/μL	6/6 – negative	5/6 – 0.1 g/l<br>1/6 – 0.3 g/l	5/6 – negative<br>1/6 – 5.6 mmol/L	6.0±0.0<br>(6/6–6.0)
2 group, control, ♂, n=6	6/6 – negative	6/6 – negative	4/6 – 0.1 g/l<br>2/6 – 0.3 g/l	6/6 – negative	6.0±0.0<br>(6/6–6.0)
3 group, GGE, ♀, n=6	3/6 – negative<br>2/6 – 10 Units/μL<br>1/6 – 25 Units/μL	6/6 – negative	4/6 – 0.1 g/l<br>2/6 – 0.3 g/l	6/6 – negative	6.0±0.0<br>(6/6–6.0)
4 group, control, ♀, n=6	6/6 – negative	6/6 – negative	4/6 – 0.1 g/l<br>2/6 – 0.3 g/l	6/6 – negative	6.0±0.0<br>(6/6–6.0)
Note: n – number of animals in a group					

Table S4: Effect of GGE on the weight of internal organs of mice with single intragastric administration at a dose of 2 g/kg of mouse body weight.

Study Group	Weight of internal organs, g						
	Brain	Heart	Lungs	Liver	Spleen	Kidneys	Gonads
1 group, GGE, ♂, n=6	0.359±0.013<br>p=0.1224	0.144±0.013<br>p=0.4724	0.178±0.010<br>p=0.3324	1.697±0.082<br>p=0.1836	0.200±0.018<br>p=0.7327	0.179±0.004<br>p=0.00002	0.0159±0.0004<br>p=0.6105
2 group, control, ♂, n=6	0.397±0.018	0.154±0.007	0.200±0.018	1.880±0.098	0.208±0.015	0.241±0.010	0.0155±0.0008
3 group, GGE, ♀, n=6	0.350±0.019<br>p=0.2464	0.151±0.001<br>p=0.7680	0.172±0.025<br>p=0.5157	1.579±0.076<br>p=0.1400	0.224±0.015<br>p=0.8493	0.153±0.005<br>p=0.0000004	0.0057±0.0005<br>p=0.7962
4 group, control, ♀, n=6	0.379±0.014	0.146±0.016	0.195±0.021	1.754±0.078	0.220±0.008	0.197±0.004	0.0059±0.0007
Note: ♂ – male symbol; ♀ – female symbol; p – significance level, $p<0.05$ – statistically significant differences compared to the corresponding values in the control group of animals; n – number of animals in the group							
